# Psychiatryai.com: An exploratory online Artificial Intelligence and Data Science platform to enable near real-time psychiatry and mental health evidence-based medical research and data dissemination

**DOI:** 10.1192/j.eurpsy.2023.1120

**Published:** 2023-07-19

**Authors:** P. Naik

**Affiliations:** Child and Adolescent Psychiatry, Central and North West London NHS Foundation Trust, Milton Keynes, United Kingdom

## Abstract

**Introduction:**

Psychiatryai.com is a prototype Artificial Intelligence (AI) and Data Science (DS) platform and research project developed for my Evidence-Based Healthcare (EBHC) course at University of Oxford in MSc studies (Kellogg College). This is a singular, multi-disciplinary, and beta-testing project in Computing Science, Psychiatry, and Mental Health for oral presentation at EPA 2023.

**Objectives:**

AI and DS in Psychiatry and Mental Health have emerged as important research areas in the post Covid-19 pandemic era. This prototype University project (Psychiatryai.com) was launched on 22nd November 2021. It aims to develop a free, secure, and open access platform in *near* real-time about psychiatry and mental health evidence-based research - for healthcare professionals, doctors, and researchers in psychiatry. The project also aims to integrate findings from the Goldacre Review (2022) into practice and develop novel computing solutions utilising AI and DS, and present findings.

**Methods:**

A WordPress site (Psychiatryai.com) was developed with syndicated RSS feeds across 330 psychiatry topics and refreshed by data servers hourly, 24 hours a day, 7 days a week. A total of 43 WordPress plugins were utilised to develop this secure platform. The site is powered by intuitive data modelling and analytics in near real-time and available in open access coding format for peer-review, future development, and research. The primary sources of live evidence for the project are PubMed and University of Helsinki, Finland. The server performance data analytics will be available for poster presentation at EPA 2023. This includes full statistical results and discussion since its inception and launch (including traffic, MESH tags, and PubMed ID), and robust technical analysis and performance outcomes - available freely online to promote research in psychiatry and mental health.

**Results:**

Knowledge Synthesis and Dissemination:

Total Words: 4356886 *

Live Psychiatry and Mental Health Citations from PubMed: Exceeds 325000

Total Evidence Alerts Published: 54391 *

Total Algorithms/Topics: 330

Total site visitors: 8023 *

* Since launch of Psychiatryai.com on 22 November 2021 inclusive to 31 October 2022

**Conclusions:**

Psychiatryai.com was able to demonstrate succesful development of an effective and viable platform to study AI and DS in Psychiatry and Mental Health, as evidenced by results table. The platform has also incorporated findings from the Goldacre Review (2022) and aims to continue to collect valuable insights towards *full* real-time data analytics and dissemination of peer-reviewed current evidence in the future. The emergence of these technologies will be useful in settings such as disaster psychiatry, psychiatry e-training and research, and e-mental health awareness/promotion ahead.

**Disclosure of Interest:**

P. Naik Consultant of: Dr Paras Naik (Psychiatryai.com) Non-profit University Project

